# Medical glove durability during exposure to different solvent agents: an ex-vivo experimental study

**DOI:** 10.1186/s13037-024-00400-4

**Published:** 2024-05-26

**Authors:** Ashley Herkins, Katrina Cornish

**Affiliations:** 1 Department of Food, Agricultural, and Biological Engineering, The Ohio State University, 590 Woody Hayes Drive, Columbus, OH 43210 USA; 2 Department of Horticulture and Crop Science, The Ohio State University, 1680 Madison Avenue, Wooster, OH 44691 USA; 3 EnergyEne Inc, 5659 Canaan Center Road, Wooster, OH 44691 USA

**Keywords:** Medical gloves, Glove durability, Latex gloves, Solvent exposure

## Abstract

**Background:**

Medical professionals are constantly exposed to bodily fluids and sanitizing agents during routine medical procedures. Unbeknownst to many healthcare workers, however, the barrier integrity of medical gloves can be altered when exposed to these substances, potentially resulting in exposure to dangerous pathogens.

**Methods:**

This experimental study was designed to test the hypothesis that the durability of both natural and synthetic solvent-exposed medical gloves will be lower than the durability of the gloves in air. The testing consisted of a sample of commercially available medical gloves exposed to 70% ethanol, phosphate buffered saline, and deionized water, aimed at simulating the environments in which medical gloves are commonly worn. Gloves were included in this study based on their performance in previous durability studies in air. Data were collected over a period of three months. The glove assessment device automatically detects pinhole-sized perforations in medical gloves, eliminating the need to visually inspect each glove. Relative durability was measured as the average number of sandpaper touches until glove puncture.

**Results:**

Four out of five glove brands performed better when exposed to all three solvents than in air, which is likely due to slippage in the interface between the wet glove and the sandpaper. Sensicare Micro, a polyisoprene surgical glove, had the most consistent durability in all three solvents tested. A two-way ANOVA revealed that both glove brand (*P* = 0.0001), solvent (*P* = 0.0001), and their interaction (*P* = 0.0040, α = 0.05) significantly affected average glove durability.

**Conclusions:**

Glove durability did not remain consistent in 70% ethanol, phosphate buffered saline, deionized water, and air. These results make it clear that additional testing and labeling information would help healthcare workers select gloves for use in specific environments to ensure the best barrier protection against disease or toxins.

## Introduction

The critical role of medical gloves in disease prevention was highlighted during the COVID-19 pandemic when the demand for these essential protective items doubled in the United States, leading to widespread shortages [1]. Previous studies have shown that glove durability varies greatly depending on glove material, type, thickness, usage time, and manufacturer, and that substandard gloves put the health of safety of healthcare workers and patients at risk [1–5].

To ensure the barrier of their gloves remains intact throughout lengthy medical procedures, surgeons commonly wear two layers of gloves, as doing so reduces the number of perforations by up to 71% compared to the use of a single pair of gloves [6]. In addition, one study found that double-gloved latex surgical gloves contained no perforations after being subjected to high-friction two-handed knot tying techniques [7]. However, these studies do not account for the fluids that medical professionals frequently encounter, such as saliva, blood, and disinfectants, that have the potential to alter the physical properties of their medical gloves and, the authors hypothesize, reduce their effectiveness as protective barriers [8].

The mechanical properties of medical gloves have been demonstrated to change depending on solvent exposure. A study on the durability of natural and synthetic medical gloves that had been exposed to phosphate buffered saline (PBS), ethanol, and air concluded that relative glove performance depended upon the solvent (if any) in which the glove had been submerged [9]. The order of failure for solvent-exposed gloves does not necessarily match the failure order of dry gloves [2, 9]. Another study demonstrated that the application of alcohol-based hand rubs to nitrile and latex examination gloves resulted in decreased tensile strength and increased ultimate elongation, particularly of the nitrile samples [10].

The objective of this study was to utilize an automated glove assessment device [11] to determine the relative durability of natural and synthetic medical gloves exposed to common medical solvents in order to simulate glove use in realistic environments.

## Materials and methods

### Gloves

The gloves selected for this study were chosen because they had been previously demonstrated to have superior durability in dry conditions [2]. Synthetic gloves included U.S. Medical Glove (nitrile examination gloves), Montgomery, IL, USA, Sensicare Neoprene (polychloroprene surgical gloves; Medline), Northfield, IL, USA, and Sensicare Micro (polyisoprene surgical gloves; Medline), Northfield, IL, USA. The natural latex gloves included Triumph Micro (latex surgical gloves; Medline), Northfield, IL, USA and Aloe Touch (latex examination gloves; Medline), Northfield, IL, USA. Glove sizes were selected to fit securely on the prosthetic hand of the Glove Assessment Device (GAD), namely, sizes medium and large or numerical sizes 6–8. Five trials were conducted for each sample/solvent combination with the exception of Sensicare Micro polyisoprene surgical gloves tested in PBS, which had four trials only due to one defective glove. Thicknesses were an average of three measurements taken at the middle finger of the glove.

### Glove Assessment device (GAD)

The Glove Assessment Device (GAD) that was used to determine relative glove durability eliminates the need to manually inspect gloves for holes, or perform a water leak test, because it relies on a vacuum within the base of a prosthetic hand, which creates a seal when a glove is donned [11]. The two middle fingers of the hand are porous, allowing airflow into the base of the hand only when a puncture occurs in the glove. A pressure sensor causes the GAD to automatically cease operation when a pressure spike occurs due to a perforation in the glove. To induce a perforation, the GAD uses a strip of 120-grit waterproof sandpaper clamped onto a mobile drum that touches the fingertips of the prosthetic hand repeatedly at a set force. The sandpaper was replaced for each new glove tested. The liquid spray functionality of the GAD was used to simulate medical environments more realistically. All settings on the GAD were set to default [11] apart from the sprayer, which was set to spray the glove fingers once between each touch of the sandpaper drum. Data were collected over a period of three months.

### Solvents

The selected solvents were deionized water, phosphate buffered saline (PBS solution, Fisher Scientific, Pittsburgh, PA, USA), and 70% ethanol solution (Fisher Scientific, Pittsburgh, PA, USA). PBS closely mimics properties of human bodily fluids, including ion concentration, osmolarity, and pH. Ethanol is commonly used as a cleaning agent and disinfectant in medical facilities. DI water was used as a reference liquid and was also used to cleanse the tubing of the GAD when changing solvents.

### Statistical analysis

The software JMP 16 was used for all statistical analyses in this study. These analyses included a two-way analysis of variance and Tukey-Kramer HSD tests. The two independent variables tested were glove type and solvent, and the independent variable was the average number of sandpaper touches until glove failure.

**Table 1:** Connecting letters report of Tukey’s HSD test of glove type. Levels not connected by the same letter are significantly different

Glove Type				Least Squares Mean
Sensicare Micro (polyisoprene, surgical)	A			637.20
Sensicare Neoprene (polychloroprene, surgical)		B		345.40
Aloe Touch (latex, exam)		B	C	206.13
Triumph Micro (latex, surgical)		B	C	188.16
U.S. Medical Glove (nitrile, exam)			C	87.47

## Results

**Fig. 1:**
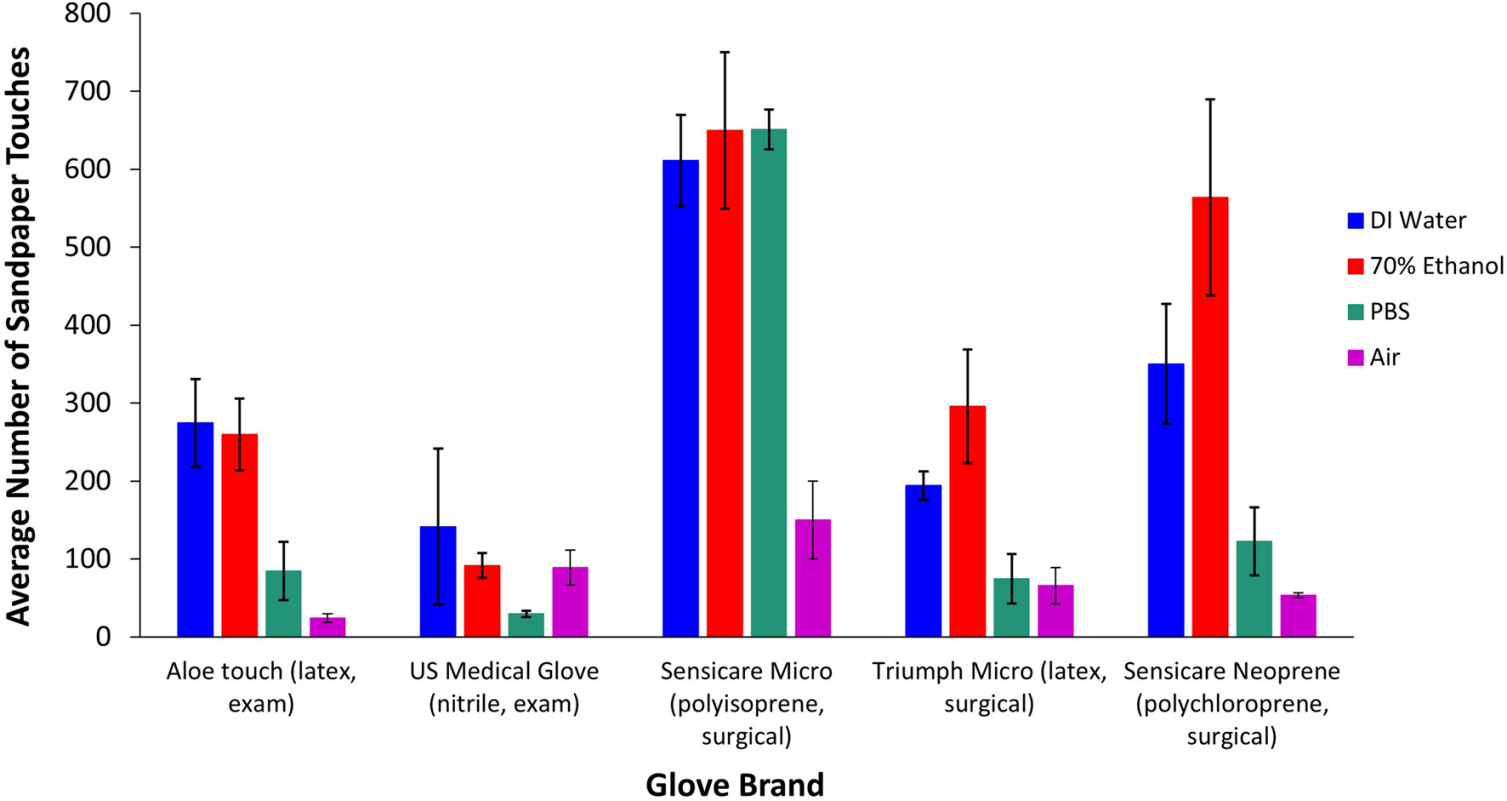
Average number of sandpaper touches until glove failure in DI water, 70% ethanol, PBS, and air *±* standard error. Values are the mean of 5 samples except for Sensicare Micro polyisoprene surgical gloves tested in PBS, which had four trials only due to a defective glove

Glove durability differed drastically depending on the type of solvent to which the glove was exposed, with the exception of Sensicare Micro brand polyisoprene surgical gloves which had a consistent performance in all three solvents (Fig. 1). Gloves exposed to solvents of any kind generally were more durable than gloves tested in air (Fig. 1). Most surgical gloves performed better than exam gloves in all three solvents; however, the Aloe Touch brand natural latex exam gloves had very similar durability to the Triumph Micro latex surgical gloves (Fig. 1). This result indicates that material plays an important role in solvent-exposed glove durability, regardless of glove usage type (surgical or exam). The U.S. Medical Glove brand nitrile exam glove was also the only glove to perform better in air than in one of the solvents, in this case PBS (Fig. 1).

The two-way ANOVA revealed that at least one factor was significant (*P* = 0.0001). The subsequent effects test showed that glove type (*P* = 0.0001), solvent (*P* = 0.0001), and their interaction (*P* = 0.0040) all significantly affected durability (average number of sandpaper touches to failure). The Sensicare Micro polyisoprene surgical glove lasted significantly longer than the other gloves under testing (Tukey’s HSD test, Table 1). Gloves tested in PBS and air had significantly different average number of touches than the other two test treatments (Table 2). The connecting letters report for the cross effect of glove type and solvent revealed that no singular glove/solvent combination resulted in a significantly different least square mean from all other combinations (Table 3).

**Table 2:** Connecting letters report of Tukey’s HSD test of solvent type. Levels not connected by the same letter are significantly different

Solvent			Least Squares Mean
70% Ethanol	A		372.08
DI Water	A		314.16
PBS		B	192.73
Air		B	81.55

**Table 3:** Connecting letters report of Tukey’s HSD test of the cross effect of glove type and solvent type. Levels not connected by the same letter are significantly different

Glove Type, Solvent						Least Squares Mean
Sensicare Micro (polyisoprene, surgical), PBS	A	B				651.0
Sensicare Micro (polyisoprene, surgical), 70% Ethanol	A					649.8
Sensicare Micro (polyisoprene, surgical), DI Water	A	B	C			610.8
Sensicare Neoprene (polychloroprene, surgical), 70% Ethanol	A	B	C	D		563.6
Sensicare Neoprene (polychloroprene, surgical), DI Water	A	B	C	D	E	350.2
Triumph Micro (latex, surgical), 70% Ethanol		B	C	D	E	295.8
Aloe Touch (latex, exam), DI Water			C	D	E	274.4
Aloe Touch (latex, exam), 70% Ethanol				D	E	259.6
Triumph Micro (latex, surgical), DI Water					E	194.0
Sensicare Micro (polyisoprene, surgical), Air					E	175.8
U.S. Medical Glove (nitrile, exam), DI Water					E	141.4
Sensicare Neoprene (polychloroprene, surgical), PBS					E	122.4
U.S. Medical Glove (nitrile, exam), 70% Ethanol					E	91.6
U.S. Medical Glove (nitrile, exam), Air					E	88.8
Aloe Touch (latex, exam), PBS					E	84.4
Triumph Micro (latex, surgical), PBS					E	74.7
Triumph Micro (latex, surgical), Air					E	65.7
Sensicare Neoprene (polychloroprene, surgical), Air					E	53.3
U.S. Medical Glove (nitrile, exam), PBS					E	29.4
Aloe Touch (latex, exam), Air					E	24.2

**Fig. 2:**
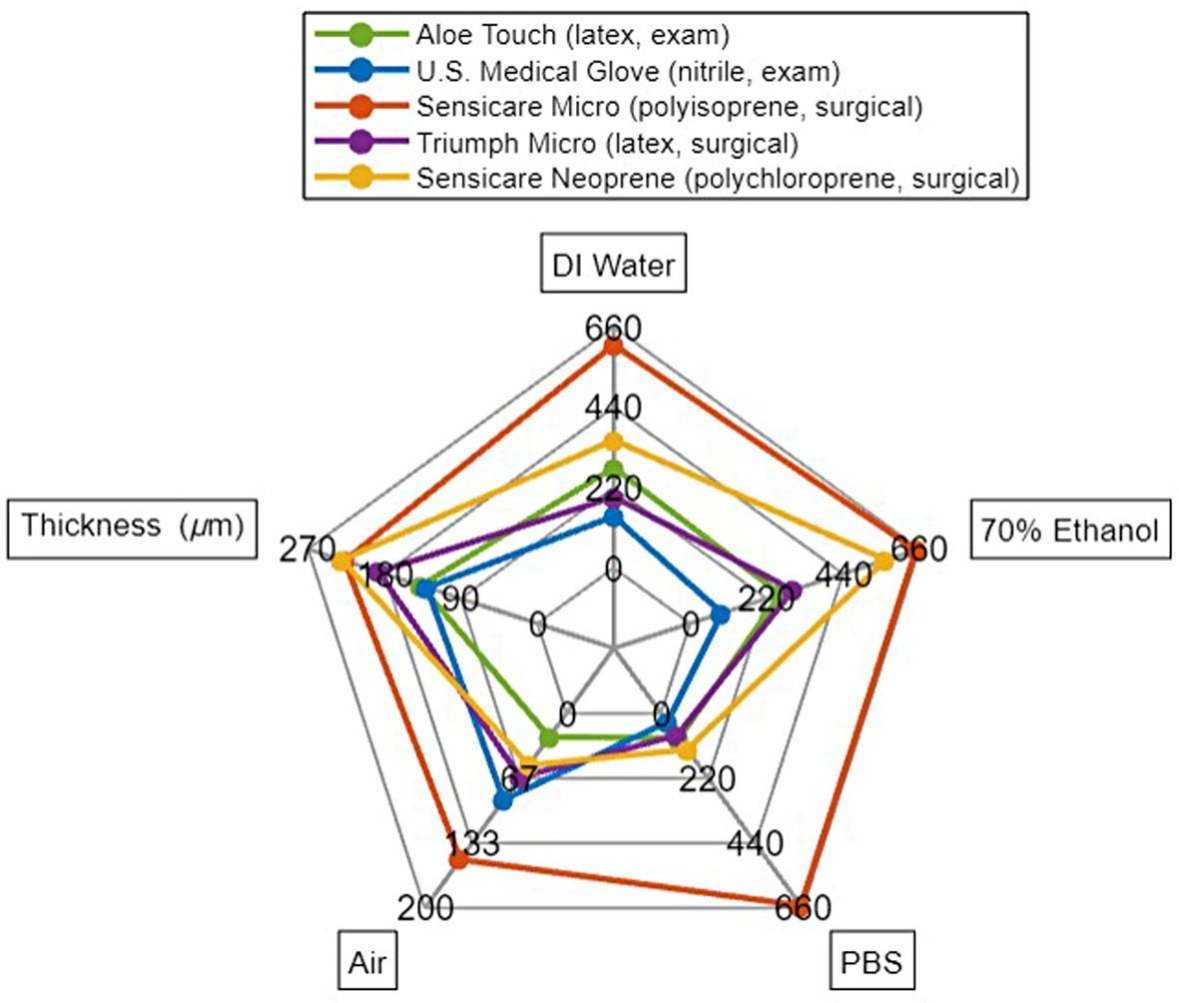
Radar plot of number of sandpaper touches to failure in DI water, 70% ethanol, PBS, and air. Glove thickness (µm) is also included for reference. Numeric labels represent the scale of the radar plot. Note: the axis for air in not the same scale as those for DI water, ethanol, and PBS.

Surgical gloves are usually thicker than examination gloves and have higher mechanical performance requirements than exam gloves (ASTM D3578-19 and D3577-19) and so are expected to have greater durability [12, 13]. However, although Sensicare Neoprene brand polychloroprene surgical gloves were the thickest gloves out of those tested, they were the second least durable in air and were less durable than the Sensicare Micro brand polyisoprene surgical gloves in all solvents (Fig. 2).

Sensicare Micro (polyisoprene, surgical) and Sensicare Neoprene (polychloroprene, surgical) brand gloves were of similar thickness, but had drastically different durability under most testing conditions (Fig. 2). Similarly, U.S. Medical Glove brand nitrile exam gloves and Aloe Touch brand latex exam gloves were the same thickness but did not have similar durability.

## Discussion

Average glove durability was generally lower in air than in any of the solvents tested. The likeliest explanation for this phenomenon is the presence of slippage within the sandpaper/glove interface when liquids are introduced. The solvents likely acted as lubricants, lessening the interaction of the abrasive sandpaper with the glove film.

ASTM International provides specific standards which all examination gloves (D3578-19) and surgical gloves (D3577-19) must meet, regardless of whether they are made of natural (Type I) or synthetic (Type II) polymers [12, 13]. Because the sampled gloves have previously been confirmed to meet these standards, substandard glove quality can be ruled out as a cause of durability differences [2].

The large variation in durability observed between gloves of similar thickness indicates that the composition of the glove, and not solely its thickness, is responsible for its performance, as has been previously concluded [2, 14]. One explanation for the gloves failing more readily in PBS than in other solvents is that PBS is hydrophobic, which may allow greater penetration into the gloves, and acting as a plasticizer. This may also account for why the U.S. Medical Glove brand nitrile exam glove performed more poorly in PBS than in air. Also, natural latex gloves contain non rubber constituents, including protein and lipids. The PBS may have extracted entrained proteins and perturbed the cured glove matrix making it less durable [15, 16].

The durability of the two natural latex gloves tested (Aloe Touch exam and Triumph Micro surgical) were more similar in the three solvents than in air because the liquid-polymer interaction is consistent in gloves made of the same material.

A significant factor in solvent permeation through medical gloves is movement, which could not be simulated by the GAD. Latex gloves have been previously demonstrated to have similar ethanol permeation rates regardless of movement [17]. In contrast, the permeation of ethanol through nitrile gloves was significantly higher when the gloves were flexed during solvent exposure [17]. Since the gloves tested on the GAD were placed on a stationary prosthetic hand, the true glove barrier effectiveness is expected to decrease when the gloves are worn on the hands of healthcare providers.

The implications of these findings are extremely important for healthcare professionals who regularly wear medical gloves, especially during surgical procedures. A glove that remains intact in air could have an entirely different barrier effectiveness when exposed to bodily fluids or sanitizing agents. Micro perforations as small as 27 nm can allow for the transfer of the smallest human pathogenic viruses resulting in the spread of healthcare-associated infections (HAIs) [18]. Therefore, it appears that additional testing and labeling information may be needed so that healthcare professionals can select gloves that provide the best barrier protection in specific environments against disease or toxins for themselves and their patients.

## Conclusions

Durability of gloves that were most durable in air is heavily influenced by solvent exposure, glove material, glove thickness, and glove usage type. During medical procedures where gloves are exposed to bodily fluids and disinfecting agents, reduced glove barrier efficiency can lead to the spread of potentially life-threatening healthcare-associated infections. Future studies should include a much larger sample of commercially available medical gloves to provide healthcare professionals with a more complete representation of relative glove performance in solvents, drugs, and air.

## Data Availability

The datasets used and/or analyzed during the current study are available from the corresponding author on reasonable request.
